# Probing the Influence of Specular Reflection and Overexposure on Backscattering Mueller Matrix Polarimetry for Tissue Imaging and Sensing

**DOI:** 10.3390/bios15050333

**Published:** 2025-05-21

**Authors:** Wei Jiao, Nan Zeng, Rui Hao, Hui Ma, Chao He, Honghui He

**Affiliations:** 1 Guangdong Research Center of Polarization Imaging and Measurement Engineering Technology, Shenzhen Key Laboratory for Minimal Invasive Medical Technologies, Institute of Biopharmaceutical and Health Engineering, Tsinghua Shenzhen International Graduate School, Tsinghua University, Shenzhen 518055, China; jiaow23@mails.tsinghua.edu.cn (W.J.); zengnan@sz.tsinghua.edu.cn (N.Z.); haor22@mails.tsinghua.edu.cn (R.H.); mahui@tsinghua.edu.cn (H.M.); 2 Department of Engineering Science, University of Oxford, Parks Road, Oxford OX1 3PJ, UK

**Keywords:** polarimetry, Mueller matrix, overexposure, tissue imaging and sensing

## Abstract

Mueller matrix polarimetry has great potential for tissue detection and clinical diagnosis due to its ability to provide rich microstructural information accurately. However, in practical in vivo tissue imaging based on backscattering Mueller matrix polarimetry, specular reflection is often inevitable, leading to overexposed regions and the following inaccurate polarization information acquisition of tissues. In this study, we probe the influence of specular reflection and overexposure on backscattering Mueller matrix polarimetry for tissue imaging and sensing. We investigate in detail the differentiation of polarization behaviors between the specular reflection and non-specular reflection tissue regions using a 3 × 3 backscattering Mueller matrix measurement. Then, we obtain the vertical projection profiles to further quantify the Mueller matrix elements of porcine liver tissue in different specular reflection regions. Finally, we calculate the polarization feature parameters derived from a 3 × 3 Mueller matrix and analyze their behavior in overexposed regions. Based on the quantitative analysis and comparisons, we obtain a group of polarization feature parameters with strong immunity to the specular reflection process. This study offers a strategy for selecting the polarization parameters during in vivo polarimetric imaging applications, provides valuable references for further eliminating the characterization errors induced by specular reflection, and may contribute to the advancement of quantitative tissue polarimetric imaging and sensing.

## 1. Introduction

Polarization techniques, due to their non-invasive, label-free, and microstructure-sensitive advantages, have shown great potential for application in biomedical imaging and sensing [1,2,3,4]. Mueller matrix (MM) imaging, as a prevalently utilized polarimetric method, has the capability to encode samples’ complete polarization properties such as diattenuation, retardance, and depolarization [5,6,7,8]. To quantitatively decode polarization-related structural characteristics of biological tissues, polarization basic parameters (PBPs) were obtained using Mueller matrix transformation (MMT), Mueller matrix polar decomposition (MMPD), and other MM analyzing approaches [9,10,11,12]. The tissue MM polarimetric sensing techniques can be categorized as backscattering MM polarimetry for imaging of bulk tissue samples and transmission MM microscopy for imaging of thin tissue slices [5,13,14]. The backscattering MM polarimetry, commonly used for in vivo tissue measurement, offers a promising sensor for various clinical applications, including skin tissue analysis [15,16], minimally invasive procedures [5], and surgical polarimetric endoscopy (SPE) [7,17,18,19].

Surgical white-light endoscopy using optical sensor allows surgeons to inspect the morphological structures of suspicious areas in detail without invasively opening and exposing the tissue, playing a crucial role in cancer and lesion detection [20,21]. By providing additional polarization information, SPE can enhance imaging contrast to better distinguish abnormal areas from normal tissues [18,22,23]. However, the precise characterization and sensing of polarization properties in SPE imaging remains a challenge. It was discovered that optical elements of rigid endoscopes can destroy the integrity of the original spectral and polarization properties [24]. The relationship of polarimetric outcomes and signal-to-noise ratio was comprehensively analyzed, demonstrating that the signal-to-noise ratio depends on both light intensity and degree of linear polarization [25]. Some researchers also investigated coordinate system selection and complex spatial illumination scheme optimization [13,26]. Although these studies improve the characterization accuracy of polarization properties for backscattering MM imaging and sensing, unexplored spaces still exist. Especially, during SPE imaging, smooth and regular biological tissue surfaces can bring prominent specular reflection, resulting in overexposed/pixel-saturated regions and the following inaccurate polarization information acquisition of tissues [18,27]. Due to the uneven surface of organ cavities, overexposure is inevitable during practical SPE imaging, leading to some methods, such as the digital image processing method, dynamic range magnification, and computer vision techniques, being ineffective [18,28,29]. Therefore, probing the influence of a large area of specular reflection and overexposure on backscattering MM polarimetry is necessary for in vivo tissue MM imaging and sensing.

Here, we investigate in detail the differentiation of polarization behavior between the specular reflection (SR) and non-specular reflection (NSR) tissue regions using a 3 × 3 backscattering MM polarimetry. The SR region is further divided into the borderline overexposed and complete overexposed regions. We measure the vertical projection profiles to quantify the MM elements of porcine liver tissue in the above three regions. Subsequently, we probe the influence of specular reflection and overexposure on polarization properties. Finally, the 3 × 3 MMT and MMPD parameters are calculated, and their differentiations are analyzed in the overexposed region, obtaining a group of parameters with strong immunity to the SR process. This study aims to offer comprehensive strategies for selecting the PBPs during SPE sensing and other practical in vivo polarimetric applications, provide valuable references for further eliminating the characterization errors induced by SR, enhance the accuracy and reliability of polarimetric measurements, and contribute to the advancement of quantitative SPE and tissue polarimetric imaging and sensing.

## 2. Materials and Methods

### 2.1. Sample and Experimental Setup

**Figure 1:**
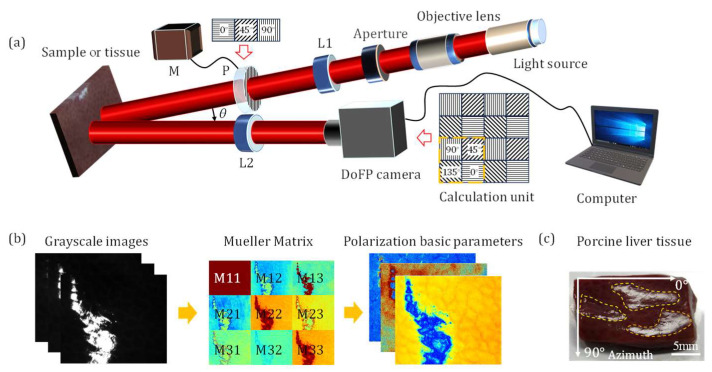
Schematic of the experimental setup and sample: (**a**) backscattering MM imaging setup using a DoFP camera. *L*_1_ and *L*_2_, lenses; *P*, polarizer; *M*, screw linear motor. (**b**) Flowchart of PBPs acquisition based on 3 × 3 MMs. (**c**) Porcine liver tissue. The SR regions are inside the yellow dashed areas, and azimuth references to the coordinate system.

In this study, a division of focal plane (DoFP) camera is employed for 3 × 3 MM imaging and sensing, suitable for in vivo backscattering tissue polarimetry [8,30]. As depicted in Figure 1a, monochromatic light emitted by the light-emitting diode (3 w, 633 nm, Δλ = 20 nm, Daheng Optic, Beijing, China) is collimated into a parallel beam by a 4× objective lens (Hengyang Optic, Guangzhou, China). It passes through an adjustable aperture (LBTKE Optic, Changsha, China) and a collimating lens *L*_1_ (LBTKE Optic, Changsha, China) to further refine the light for improved imaging quality. The collimated beam is sequentially modulated by three fixed polarizers *P* (extinction ratio > 1000:1, LBTKE Optic, Changsha, China), which is driven by a screw linear motor *M* (FSK30, FUYU Technology, Chengdu, China), to generate 0°, 45°, and 90° linear states of polarization illuminations in series during each measurement. After passing through the imaging objective lens *L*_2_ (Hengyang Optic, Guangzhou, China), the scattered photons from the tissue sample are precisely collected by the DoFP camera (PHX050S-P, Lucid Vision Labs, Richmond, BC, Canada).

The DoFP camera contains numerous calculation units (inside the yellow dashed square, as shown in Figure 1a) capable of autonomously capturing intensity information at four different polarization angles within different emergent light. The camera quantifies and encodes the information into grayscale numerical data with different polarization states before transmitting it to the computer. As shown in Figure 1b, we can construct MMs of the tissue samples from the grayscale images and extract corresponding PBPs to directly characterize polarization-related microstructural information. In this study, we select porcine liver tissue as the specimen for MM measurement due to its smooth surface, rich polarization properties, and well-defined boundary, which produces a distinct specular reflection phenomenon, as indicated by the yellow dashed areas in Figure 1c. The animal experimentation work has been approved by the Ethics Committee of Tsinghua Shenzhen International Graduate School, Tsinghua University.

Before conducting measurements, each optical component has been calibrated using a polarimeter (PAX1000, Thorlabs, Newton, NJ, USA) to ensure that the systematic errors are within 1%. During the measurement process, a constant distance is rigorously maintained between DoFP and the tissue surface to minimize errors from illumination intensity variations or focal length. Also, the angle between the polarization state generator (PSG) arm and the polarization state analyzer (PSA) arm is maintained at 10° to prevent polarization aberration induced by complex spatial illumination [31]. Here, we modulate the specular reflection using a coverslip. Specifically, when a coverslip is used with an incident angle of 10° and an emergent angle of 0°, no specular reflection occurs. When measured without a coverslip, due to wrinkles on the surface of porcine liver tissue, the incident light angles on the local surface range randomly between 0° and 10°, with regions where the emergent angle matches the incident angle. The glass coverslip does not introduce additional polarization effects [32]. The 3 × 3 MMs of the sample are reconstructed according to Equations (1) and (2), where *DoFP*, *L*_2_, *P*, and *L*_1_ respectively represent the 3 × 3 MMs of the corresponding optical components, and *S_in_* and *S_out_* represent the input and output Stokes vectors [13].

(1) $$S_{out}=DoFP\times L_{2}\times MM_{S}\times P\times L_{1}\times S_{in},$$

(2) $$MM_{S}=DoFP^{-1}\times S_{out}\times {S_{in}}^{-1}\times P^{-1}.$$

**Figure 2:**
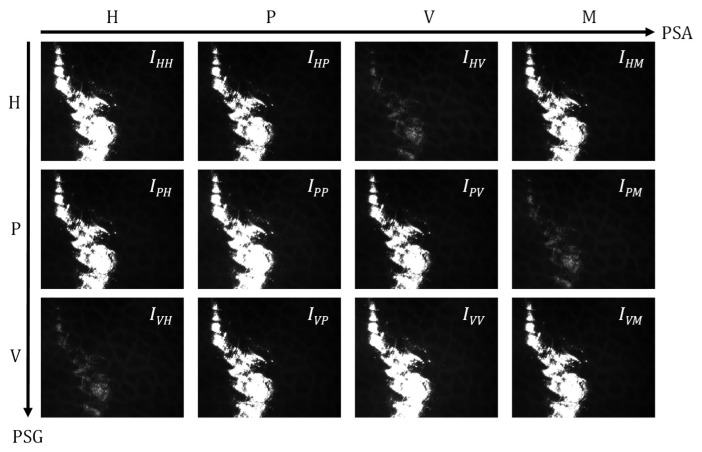
Grayscale images of porcine liver tissue sample with single acquisition: these sequenced grayscale images are generated by producing three polarization states for the incident light: horizontal linear (H), 45° linear (P), and vertical linear (V), and, respectively, detecting four polarization components of the emergent light: horizontal linear (H), 45° linear (P), vertical linear (V), and 135° linear (M). The first and second letters indicate the input and output polarization states, respectively. For instance, *HP* denotes horizontal linear input polarization and 45° linear output polarization.

We generated three polarization states for the incident light: horizontal linear (H), 45° linear (P), and vertical linear (V). For each incident light, we separately received four polarization state components of the scattered or reflected light from the sample based on the DoFP camera: horizontal linear (H), 45° linear (P), vertical linear (V), and 135° linear (M). Figure 2 depicts the collection of all 12 grayscale images of a porcine liver tissue specimen without a coverslip containing the SR regions. It is evident from Figure 2 that during specular reflection, most photons may retain their original polarization properties, leading to a prominent reflection elimination effect of cross-polarization measurement. Further, we can acquire the 3 × 3 MM to derive all linear polarization properties of the tissue based on the grayscale images (*I_HH_*, *I_HP_*, *I_HV_*, …, *I_VM_*).

### 2.2. Mueller Matrix Transformation (MMT) and Mueller Matrix Polar Decomposition (MMPD) Parameters

Mueller matrix encodes a comprehensive description of the polarization characteristics of media. However, individual MM elements lack explicit physical meanings or a distinct correlation with microstructures, and many polarization properties are complexly associated with multiple MM elements [5]. Therefore, several MM decomposition approaches have been proposed to quantitatively extract the optical and structural properties of a specimen. Here, we employ the MMPD and MMT methods, which are prevalently used in tissue polarimetry, to analyze the acquired 3 × 3 MMs [9,33].

The MMT parameters capable of characterizing structural characteristics can be expressed as straightforward analytical functions of the MM elements. Notably, some of the MMT parameters do not rely on full MM elements and can thus be applied to 3 × 3 MM without any modifications. The commonly utilized parameters, such as *t*, *A*, *b*, and *b*_2_, have demonstrated significant potential in pathological diagnosis and clinical application [34]. In addition, MMT offers more derivative parameters suitable for various applications, including $\tilde{b}$, $\tilde{\beta }$, $\left|B\right|$, $\left\|B\right\|$, *D*_L_, *P*_L_, and α_D_. The specific physical meanings of the aforementioned parameters can be found in previous studies [35]. Meanwhile, Table 1 gives the calculation expressions of the adopted MMT parameters, where *Mij* indicates the element in the i-th row and j-th column.

The MMPD method proposed by Lu and Chipman has been widely used in biomedical studies [10]. The 3 × 3 MMPD is essentially similar to the 4 × 4 implementation, where the Mueller matrix *M* is decomposed into three submatrices, representing depolarizer (*M*_Δ_), retarder (*M_R_*), and diattenuator (*M_D_*), as shown in Equation (3) [33,36].

(3) $$M=M_{\Delta }M_{R}M_{D},$$

Here, *M_D_* is obtained from the first row of *M* to calculate the linear diattenuation parameter *D*. A matrix *M_DR_* is constructed for obtaining *M*_Δ_ as shown in Equations (4) and (5). By calculating the maximum non-unit eigenvalue λ of *M_DR_*, the linear depolarization coefficient $\Delta =1-\sqrt{\lambda }$ can be acquired.

(4) $$M_{DR}=(M\cdot M_{D}^{-1})\cdot {\left(M\cdot M_{D}^{-1}\right)}^{T}.$$

(5) $$M_{\Delta }=\left[\begin{array}{ccc} 1 & 0 & 0\\ 0 & \sqrt{\lambda } & 0\\ 0 & 0 & \sqrt{\lambda } \end{array}\right].$$

Then, *M_R_* can be calculated as

(6) $$M_{R}=M_{\Delta }^{-1}\cdot M\cdot M_{D}^{-1}.$$

Similarly to the 4 × 4 MMPD, the *M_R_* can be decomposed into the following matrices,

(7) $$M_{R}=\left[\begin{array}{ccc} 1 & 0 & 0\\ 0 & \cos^{2}2\theta +\sin^{2}2\theta \cos\delta & \sin2\theta \cos2\theta (1-\cos\delta )\\ 0 & \sin2\theta \cos2\theta (1-\cos\delta ) & \sin^{2}2\theta +\cos^{2}2\theta \cos\delta \end{array}\right]\cdot \left[\begin{array}{ccc} 1 & 0 & 0\\ 0 & \cos2\psi & \sin2\psi \\ 0 & -\sin2\psi & \cos2\psi \end{array}\right].$$

where *δ*, *θ*, and *ψ* are the linear retardance, fast-axis orientation, and optical rotation, respectively. The specific parameter calculation expressions can be found in Table 1. The ε*_ijk_* is the Levi-Civita symbol in the *θ* calculated formula [37].

### 2.3. Analysis Methods

To visually demonstrate the variation trends of Mueller matrix with different regions, we calculate the vertical projection profile of all element images, which compresses the image into a single row by averaging across all rows [38].

(8) $$change\;rate=\frac{PBP_{SR}-PBP_{NSR}}{PBP_{SR}}.$$

Meanwhile, to evaluate the influence of SR on different parameters, we calculate the average change rate as Equation (8) shows, where *PBP_SR_* and *PBP_NSR_* are the average values of PBP from the SR and NSR regions, respectively. We utilize the *I_HH_* grayscale images to determine the SR and NSR regions by setting a grayscale threshold, and a logical mask image can be generated through binary segmentation. We can assess the robustness of different PBPs under the influence of SR by calculating the average change rate, which indicates the parameter value in SR regions with an increase or decrease compared to the value in the NSR regions.

## 3. Results

### 3.1. Influence of Specular Reflection on MM Elements

**Figure 3:**
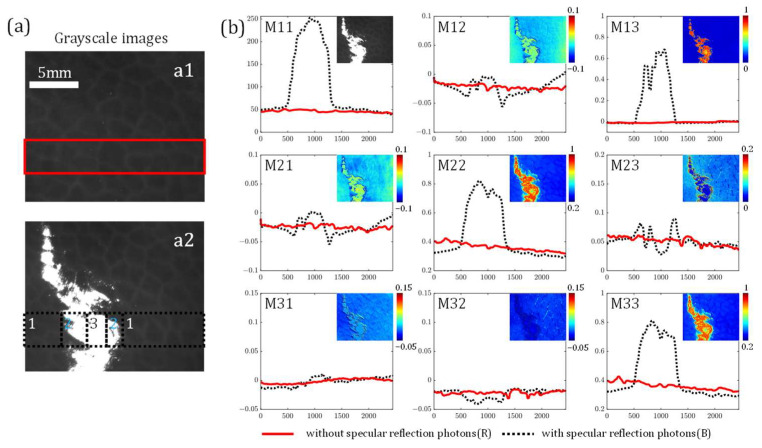
Grayscale images and vertical projection profiles of MM elements of porcine liver tissue: (**a**) grayscale images of porcine liver tissue, including (**a1**) regions with NSR, and (**a2**) regions with SR. The rectangular regions are selected to calculate the vertical projection profiles. The numbers 1, 2, and 3 in (**a2**) represent the NSR regions, borderline overexposed regions, and completely overexposed regions, respectively. (**b**) Vertical projection profiles of the corresponding regions of the MM elements. The upper right subfigures show the pseudo-color MM image. All curves are normalized by the M11.

We first analyze the influence of overexposure induced by specular reflection (SR) on individual MM elements, as Figure 3 shows. The MM images of porcine liver tissue are measured with oblique incidence and normal detection to maintain the same spatial illumination angle at 10° as illustrated in Figure 1, with the camera exposure time being 2 × 10^5^ μs. Figure 3a displays the captured grayscale image, where the SR regions can be clearly observed. Figure 3b provides the vertical projection profiles of the MM elements, where the red solid lines and the black dashed lines represent the MM elements from Figure 3(a1,a2). The horizontal axis denotes pixel position in the grayscale image (2448 points), while the vertical axis represents corresponding element values normalized by the M11.

The variation trends of MM elements clearly illustrate their polarization response to the overexposure induced by specular reflection. From the images divided into three regions, as Figure 3(a2) shows, we can conclude the following: (i) Zone 1 is manifested as the non-specular reflection regions, whose MM elements images only contain information from the porcine liver tissue carried by backscattered photons. Since the liver tissue is mostly isotropic with hepatic lobule regions, the red solid lines and black dashed lines are all close in Zone 1 as shown in Figure 3b [13]. Meanwhile, the non-diagonal element values (e.g., M12, M13, M21, M23, M31, and M32) are close to 0 with a variation of less than 0.05. On the contrary, the diagonal elements (e.g., M22 and M33) values are close to 0.4, exhibiting a significant linear depolarization property; (ii) Zone 2 shows the borderline overexposed regions, as approximately 1 mm inward from the overexposed boundary. In these regions, the MM element images contain information from both the porcine liver tissue and the surface SR photons. We can observe from the black dashed lines in Figure 3b that the non-diagonal elements present differential changes in Zone 2: the M12 and M21 show two sharp troughs; the M13 increases from 0 to 0.4 with a large gradient, and the M31 keeps close to 0, consistent with that in Zone 1, leading to a symmetry-breaking phenomenon for the M13 and M31 pair; the M23 and M32 exist subtle and disordered variations around 0. The diagonal elements M22 and M33 identically show the increase from 0.4 to 0.6, noting that the two elements exhibit the same variation with the intensity increase; (iii) Zone 3 shows the completely overexposed regions, where the MM elements contain the information mostly from the surface SR photons. It can be noticed that the non-diagonal element values in Zone 3 are close to 0, except for the M13, representing a significant magnification. The diagonal element values rise to 0.8, showing a larger linear polarizance in Zone 3.

Interestingly, when the light intensity reaches the max at the horizontal axis location x = 1000, the M22 and M33 have a locally minimum value. To explain such variations in Zone 3, we theoretically calculate the MM elements. In Zone 3, according to Figure 2, we can observe that grayscale images were obtained with almost saturated grayscale values *I_saturated_*, leading to the inaccurate expression of grayscale values, except for *I_HV_*, *I_PM_*, and *I_VH_*. Assuming the intensity values of *I_HV_*, *I_PM_*, and *I_VH_* are accurately represented by $I_{saturated}-\eta$, the theoretical result can be calculated through Equation (2) as

(9) $$\begin{array}{cc} MM_{S} & =S_{out}\times S_{in}^{-1}\\ & =\left[\begin{array}{ccc} 2I_{saturated}-\eta & 2I_{saturated} & 2I_{saturated}-\eta \\ \eta & 0 & -\eta \\ 0 & \eta & 0 \end{array}\right]\times \left[\begin{array}{ccc} 1/2 & 1/2 & -1/2\\ 0 & 0 & 1\\ 1/2 & -1/2 & -1/2 \end{array}\right].\\ & =\left[\begin{array}{ccc} 2I_{saturated}-\eta & 0 & \eta \\ 0 & \eta & 0\\ 0 & 0 & \eta \end{array}\right] \end{array}.$$

It should be noted that the backscattering imaging coordinate system is modified to keep the elements positive [26]. According to Equation (9), the M13 has a significant magnification under overexposure. When the intensity increases, as the M11 shows, *η* decreases, leading to the M22 and M33 having the locally minimum values.

### 3.2. Polarization Measurement Errors Under Overexposure Induced by Specular Reflection

**Figure 4:**
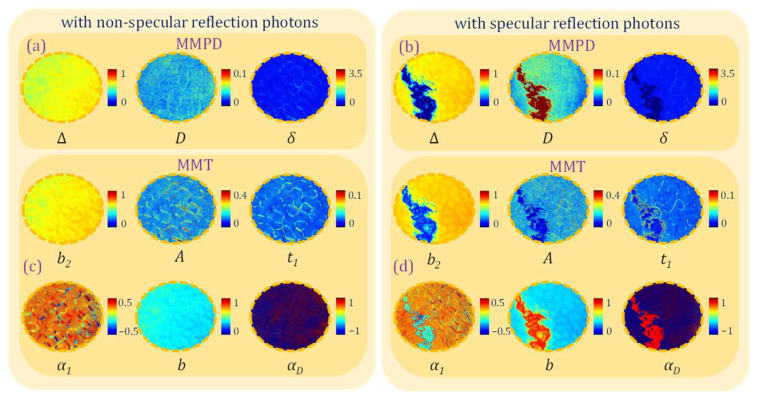
Pseudo-color PBP images of porcine liver tissue: (**a**) MMPD parameters images with NSR regions; (**b**) MMPD parameters images with SR regions; (**c**) MMT parameters images with NSR regions; (**d**) MMT parameters images with SR regions.

Polarization basic parameters (PBPs), decoding the samples’ optical and structural information from a Mueller matrix, can be inaccurate under the SR influence. Deriving from 3 × 3 MMPD and MMT, the PBPs with explicit physical meanings are calculated in both SR and NSR regions as shown in Figure 4 [34,36]. Here, the azimuthal parameters are measured in radians; all the non-azimuthal parameters are dimensionless. We use the coordinate system that aligns with Figure 1c.

The 3 × 3 MMPD parameters Δ, *D*, and *δ* images are shown in Figure 4a,b, representing linear depolarization, linear diattenuation, and linear retardance, respectively. The PBPs are also separately analyzed in Zones 1–3: (i) In Zone 1, the parameter Δ value is close to 0.6, exhibiting the obvious linear depolarization of porcine liver tissue due to its complex scattering structures. The *D* and *δ* values are close to 0, showing the sample’s dominant isotropic properties. (ii) In Zone 2, the MMPD parameter values all fall between the measured values in Zone 1 and Zone 3, averaging from the backscattering photons and SR photons; (iii) In Zone 3, we can notice that the Δ value is close to 0, exhibiting a significant false-negative linear depolarization. The parameter *D* value in Zone 3 has a significant increase, displaying noticeable false-positive linear diattenuation due to the increase in M13 led by SR. The parameter *δ* value is close to 0, showing a non-existent linear retardance.

We also calculate the MMT parameters *b*_2_, *A*, *t*_1_, *b*, *α*_1_, and *α*_D_ images as shown in Figure 4c,d, representing linear depolarization, normalized linear anisotropy, linear anisotropy, linear polarizance, anisotropic azimuth, and diattenuation azimuth, respectively. The following can be observed: (i) In Zone 1, the *b*_2_ and *b* images show the linear depolarization degrees close to 0.6. The parameters *A* and *t*_1_ images show lower values in the isotropic hepatic lobule regions than those in anisotropic connective tissue [13]. The *α*_1_ image here in Zone 1 can represent the azimuth of anisotropic connective tissue areas accurately. (ii) In Zone 2, the MMT parameter values also fall between the values in Zone 1 and Zone 3, similar to the MMPD parameters. (iii) In Zone 3, the parameter *b*_2_ value is close to 0.2, exhibiting similar false-negative linear depolarization as that in the Δ image. *A* and *t*_1_ parameter values are close to 0, indicating a significant isotropic property here, consistent with the features of the tissue sample but inconsistent with that of MMPD parameter *D*, suggesting that parameter *D* may be numerically unstable for strongly reflective media. The *α*_1_, *b*, and *α*_D_ parameter values are close to 0, 0.8, and 0.8, respectively, introducing obvious inaccuracy of polarimetric characterization here in SR regions.

By analyzing the 3 × 3 MMPD and MMT parameters images as shown in Figure 4, we can conclude that the polarization properties derived from an overexposed MM can be shown as a significant false-negative depolarization and a false-positive diattenuation, introduced by the SR processes. It means that when measuring PBPs of tissues with smooth surfaces or mucus, potential misinterpretation based on depolarization and diattenuation may happen.

### 3.3. Polarimetric Parameters Selection Strategies Under Overexposure

**Figure 5:**
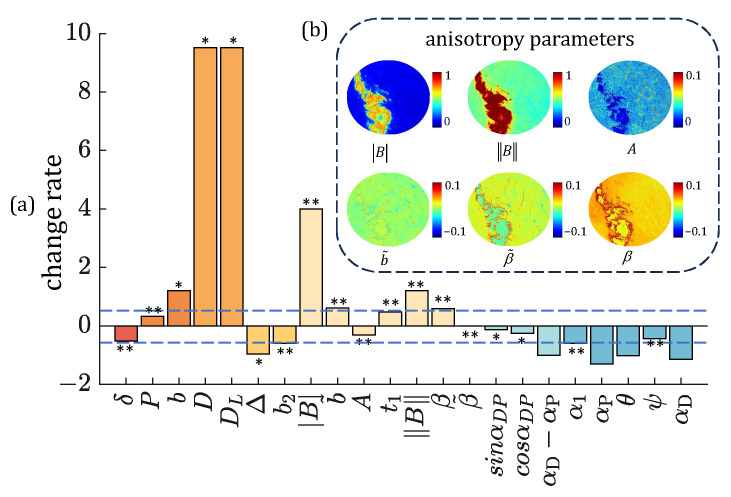
PBPs images and corresponding average change rates of porcine liver tissue: (**a**) The average change rate for 22 PBPs. ** indicates the variance less than 0.1, * indicates the variance between 0.1 and 1, and no mark indicates the variance larger than 1. The same column color represents a similar polarization property. The blue dashed lines denote the 0.5 and −0.5 scales. (**b**) Pseudo-color anisotropy-related PBPs images.

Previously, we found that some of the PBPs obtained from certain MM elements may possess better immunity to oblique incidence-induced polarimetric aberration [31]. Similarly, PBPs may also exhibit different immunities to the SR process. For a quantitative comparison, a binary mask is generated to separate Zone 3 (with the SR process) from Zone 1 and Zone 2, used to calculate the means and variances of the PBPs accordingly. Furthermore, an average change rate of each PBP is calculated using Equation (8). In Figure 5a, the PBPs representing similar polarization properties are illustrated in the same color columns. Considering the existence of oblique incidence induced by uneven tissue surfaces, an absolute change rate within 0.5 is considered a non-significant variation for the SR process, as shown within the blue dashed lines [31].

The depolarization-related PBPs *P*, *b*, Δ, and *b*_2_ can characterize the degree of photon scattering-induced randomization in backscattering measurements. For the parameters *P* and *b* images in Zone 3, they show larger linear polarizance values than those in Zone 1, with *P* parameter showing a smaller change rate and variance compared to *b* parameter. Parameters Δ and *b*_2_, denoted as linear depolarization property, both show a decline under the SR process, with *b*_2_ parameter showing a smaller change rate and variance compared to Δ parameter. The retardance-related PBPs can represent the dominant anisotropic properties in backscattering measurements [5]. Here, the linear retardance parameter *δ* obtained through the MMPD approach is compared with the anisotropy MMT parameters *A*, $\left|B\right|$, $\left\|B\right\|$, $\tilde{b}$, $\tilde{\beta }$, and *β* shown in Figure 5b [36]. It can be noticed that both $\left|B\right|$ and $\left\|B\right\|$ parameters representing linear diattenuation and linear retardance show a false anisotropy increase in the SR process, due to the effect of increasing M22 and M33 values in Zone 3. Parameter *A* and $\tilde{b}$ are close to 0 and fail to effectively provide imaging contrast. Among the above retardance-related PBPs, the normalized linear anisotropy parameter *A* shows the smallest change rate and variance in Zone 3. The change rates of parameters *D* and *D*_L_ show significant false-positive diattenuation. Meanwhile, among the anisotropic azimuth PBPs *α*_1_, *α*_P_, *θ*, *ψ*, and *α*_D_, the optical rotation angle *ψ* represents the smallest change rate and variance in Zone 3. Among the breaking of transpose symmetry information related parameters sinα_DP_, cosα_DP_, and α_D_−α_P_, the sin*α*_DP_ shows the smallest change rate and variance in Zone 3. In summary, since a larger change rate and variances indicate a distinction between PBPs in Zone 1 and Zone 3, for the strong specular reflection tissue, we should preferentially use polarization parameters *P*, *b*_2_, *A*, *ψ*, and sin*α*_DP_, with strong immunity to specular reflection.

## 4. Discussion

We probe the influence of specular reflection and overexposure on backscattering Mueller matrix polarimetry for tissue imaging and sensing. Although the primary experiments were conducted using porcine liver tissues, our findings are broadly applicable to most internal organ tissues for two main reasons. First, the MMs observed under specular reflection are primarily determined by the inherent polarization properties of specular reflection and the inaccurate representation of light intensity across different polarization channels, rather than the intrinsic polarization properties of the biological tissue. Second, our results demonstrate that diattenuation and depolarization are significantly influenced by specular reflection. Previous studies have shown that porcine liver tissue exhibits similar polarization properties to the most various internal organ tissues, exhibiting strong depolarization and weak diattenuation [5,31]. Several exceptions, such as skeletal and myocardial muscles having a strong diattenuation [5], may not be applicable to the conclusions. Consequently, the conclusions presented here are generally applicable to most types of biological tissues.

## 5. Conclusions

In this study, we investigated in detail the influence of specular reflection and overexposure on backscattering Mueller matrix polarimetry and sensing. We measured the backscattering 3 × 3 MMs of porcine liver samples. The specular reflection region in the sample was further divided into the borderline overexposed and complete overexposed regions. Subsequently, we calculated the vertical projection profiles to quantify the MM elements of porcine liver tissue in different regions. It was found that the overexposure effect can lead to significant increases in M13, M22, and M33 elements, while other elements tend to be close to 0. The MMT and MMPD parameters images indicated that the polarization properties of samples would be destroyed in the overexposed regions, manifested as significant false-negative depolarization and false-positive diattenuation. Finally, we calculated 22 PBPs derived from 3 × 3 MMT and MMPD approaches and analyzed their differentiations in the overexposed region. By comparing the PBPs’ average change rates, we obtained a group of parameters with strong immunity to the SR process. This study offered a strategy for selecting the appropriate PBPs during practical in vivo polarimetric applications such as SPE imaging and sensing, provided valuable references for further eliminating the characterization errors induced by specular reflection, and contributed to the advancement of quantitative tissue polarimetric imaging and sensing.

## Figures and Tables

**Table 1:** Calculation expressions of 3 × 3 PBPs.

3 × 3 Mueller matrix transformation (MMT) parameters	
$b=\frac{1}{2}(M22+M33)$	$b_{2}=1-\frac{1}{2}(M22+M33)$
$t_{1}=\frac{1}{2}\sqrt{{\left(M22-M33\right)}^{2}+{\left(M23+M32\right)}^{2}}$	$A=\frac{2\cdot b\cdot t_{1}}{b^{2}+t_{1}^{2}}$
$\beta =\frac{1}{2}(M23-M32)$	$\tilde{b}=\frac{1}{2}(M22-M33)$
$\tilde{\beta }=\frac{1}{2}(M23+M32)$	$\left|B\right|=M22\cdot M33-M23\cdot M32$
$\left\|B\right\|=\sqrt{M22^{2}+M23^{2}+M32^{2}+M33^{2}}$	$\alpha_{1}=\frac{1}{4}\mathrm{atan}2(M23^{\prime }+M32^{\prime },M22^{\prime }-M33^{\prime })$
$D_{L}=\sqrt{M12^{2}+M13^{2}}$	$P_{L}=\sqrt{M21^{2}+M31^{2}}$
$\alpha_{D}=\frac{1}{2}\mathrm{atan}2(M13^{\prime },M12^{\prime })$	$\alpha_{P}=\frac{1}{2}\mathrm{atan}2(M31^{\prime },M21^{\prime })$
$\alpha_{D}-\alpha_{P}$	$\cos\alpha_{DP}=(M12\cdot M21+M13\cdot M31)/(D_{L}\cdot P_{L})$
$\sin\alpha_{DP}=(M12\cdot M31-M21\cdot M13)/(D_{L}\cdot P_{L})$	$\sqrt{{\left(M12-M21\right)}^{2}+{\left(M13-M31\right)}^{2}}$
3 × 3 Mueller matrix polar decomposition (MMPD)	
$D=\sqrt{M12^{2}+M13^{2}}$	$\delta =\arccos(\sqrt{{\left(M_{R}22+M_{R}33\right)}^{2}+{\left(M_{R}32-M_{R}23\right)}^{2}}-1)$
$\psi =\frac{1}{2}\arctan(\frac{M_{R}23-M_{R}32}{M_{R}22+M_{R}33})$	$\theta =\frac{1}{2}\arctan(\frac{r_{1}}{r_{2}}),s.t.r_{i}=\frac{1}{2\sin\delta }\times \sum_{j,k}^{3}\varepsilon_{ijk}M_{R}jk$

## Data Availability

Data underlying the results presented in this paper are not publicly available at this time but may be obtained from the authors upon reasonable request.
